# Dietary Intake of Branched Chain Amino Acids and Breast Cancer Risk in the NHS and NHS II Prospective Cohorts

**DOI:** 10.1093/jncics/pkab032

**Published:** 2021-04-12

**Authors:** Deirdre K Tobias, Boyang Chai, Rulla M Tamimi, JoAnn E Manson, Frank B Hu, Walter C Willett, A Heather Eliassen

**Affiliations:** 1 Division of Preventive Medicine, Department of Medicine, Brigham and Women’s Hospital and Harvard Medical School, Boston, MA, USA; 2 Department of Nutrition, Harvard T.H. Chan School of Public Health, Boston, MA, USA; 3 Channing Division of Network Medicine, Department of Medicine, Brigham and Women’s Hospital, Boston, MA, USA; 4 Department of Epidemiology, Harvard T.H. Chan School of Public Health, Boston, MA, USA

## Abstract

**Background:**

Branched chain amino acids (BCAAs) are essential amino acids common throughout the US diet. Although circulating BCAAs have been implicated in insulin resistance and some obesity-related cancers, the relationship between dietary intake of BCAAs and incident breast cancer is unknown. We sought to evaluate the association between long-term dietary intakes of BCAAs and invasive breast cancer risk.

**Methods:**

Our analyses included 196 161 women from the Nurses’ Health Study and Nurses’ Health Study II longitudinal cohorts. Average intakes of total and individual BCAAs (isoleucine, leucine, valine) were estimated from repeated diet questionnaires and incident self-reported breast cancer cases were confirmed via medical record review. Cox proportional hazards models, adjusted for reproductive history, lifestyle, body mass index, and other breast cancer risk factors, were used to estimate hazard ratios and 95% confidence intervals.

**Results:**

We observed 10 046 incident cases of breast cancer over a median of 20.8 years of follow-up. No associations between dietary intakes of total or individual BCAAs with breast cancer risk were observed. Compared with women in the bottom quintile of BCAA intake, the hazard ratio of breast cancer for those in the top quintile was 1.05 (95% confidence interval = 0.98 to 1.12; 2-sided *P*_trend_ = .20). Findings were consistent across molecular subtypes and according to type 2 diabetes diagnosis and body mass index categories.

**Conclusions:**

Dietary intakes of BCAAs are not likely a risk factor for breast cancer.

Branched chain amino acids (BCAAs; isoleucine, leucine, and valine) are essential amino acids derived solely from diet and from a wide range animal and vegetable proteins, including processed meats, fish, poultry, dairy, beans, and some grains. The metabolism of BCAAs as a building block in protein synthesis is well characterized, contributing to a wide range of physiologic functions throughout the human body (1). However, recent metabolomics studies identified circulating BCAA levels as positively correlated with obesity and markers of impaired carbohydrate metabolism and are independent predictors of type 2 diabetes and cardiovascular disease endpoints (2,3). Circulating BCAAs have also been implicated in metabolomic studies of some insulin resistance–related cancers, including pancreatic cancer and most recently postmenopausal breast cancer (4,5). Previous observational evidence suggests heterogeneity across sources of dietary protein intakes with breast cancer risk (6). For example, in a prior analysis in the Nurses’ Health Study (NHS) II, women in the highest vs lowest quintiles of long-term red meat intake had a 22% higher risk of breast cancer (7). However, Cox proportional hazards regression models observed that modeling a 1-serving-per-day decrease of red meat and a 1-serving-per-day increase in legumes or poultry to approximate a substitution between protein sources found 15% and 17% lower breast cancer risks, respectively. In addition, given the correlation between dietary BCAAs with circulating BCAAs is typically low, with correlation coefficients less than 0.2 (8), it is unknown whether long-term intake of BCAAs per se would be associated with breast cancer risk.

Previous mechanistic evidence from basic human and animal studies are largely consistent, demonstrating BCAAs have the ability to impair insulin action and signaling in skeletal muscle through upregulation of the mTOR pathway (9-14). Breast cancer is another cancer site with an obesity and insulin resistance link, and a plausible role for BCAAs was recently strengthened by compelling research demonstrating leucine’s impact on cell proliferation and treatment resistance in estrogen receptor-positive (ER+) breast cancer cells (15). However, despite promising research of potential mechanisms and epidemiologic studies of circulating metabolites, little is known about the role of dietary intakes of BCAAs in relation to breast cancer incidence. Further, the correlations between dietary intakes of BCAAs with concentrations in circulation are only modest, suggesting the relationship of metabolites with incident breast cancer may differ from that of diet and cancer.

Therefore, we evaluated dietary intakes of BCAAs in relation to breast cancer risk in 2 large prospective cohorts of US women. The NHS and NHS II have repeated measures of diet spanning several decades of follow-up. Further, incident breast cancer cases are confirmed, and detailed information on tumor characteristics is collected. We hypothesized that dietary intakes of BCAAs confer a modest increased risk of incident breast cancer.

## Methods

### Study Population

We conducted a prospective longitudinal analysis of dietary BCAA intake in relation to incident breast cancer risk in the NHS and NHS II cohorts. The NHS and NHS II were established in 1976 and 1989 with 121 701 and 116 429 female nurses, respectively. NHS participants were aged 30-55 years and NHS II participants were 25-42 years at study baseline. Baseline questionnaires were administered in both cohorts to establish medical and reproductive history, lifestyle characteristics, and other factors. Questionnaires update this information on a biennial basis. A semiquantitative food frequency questionnaire (FFQ) was also administered to the NHS with 61 items in 1980 and NHS II with 116 items in 1991, which were expanded to include 152 items. The FFQs are administered to update usual diet approximately every 4 years. The study protocol was approved by the institutional review boards of the Brigham and Women’s Hospital and Harvard T.H. Chan School of Public Health as well as those of participating registries as required.

### Diet Assessment

Diet assessment via FFQs was conducted every 4 years to ascertain usual intake of foods and beverages over the past 12 months. The nutrient content of items, including protein and amino acid intakes, was derived according to the US Department of Agriculture database, food manufacturer data, and other published resources. A previous validation study compared total protein intake estimates derived from FFQ against repeated 7-day diet records and demonstrated good validity in capturing types of protein intake: Spearman correlation *r* = 0.56 for animal protein; *r* = 0.66 for vegetable protein (16).

### Breast Cancer Case Ascertainment

The NHS and NHS II biennial questionnaires captured incident disease diagnoses, including breast cancer. Self-reported cases of cancer were confirmed via participants’ medical records by a committee of physicians with 99% accuracy, and information was extracted for invasive vs in situ, hormone receptor status, and tumor characteristics, when available (94% of cases) (17). Deaths were identified by the postal service, next of kin, or National Death Index, with medical records or death certificates used for additional documentation of breast cancer. Our analyses included first primary invasive breast cancers reported through June 2014 (NHS) or June 2015 (NHS II).

ER, progesterone receptor (PR), human epidermal growth factor receptor 2 (HER2), cytokeratins (CK5/6), epidermal growth factor receptor (EGFR), and tumor grade were used to classify tumors into molecular subtypes. Cases that were ER+ and/or PR+ and HER2− and histologic grade 1 and 2 were classified as luminal A cancers; cases that were either: 1) ER+ and/or PR+ and HER2+, or 2) ER+ and/or PR+, HER2−, and histologic grade 3 were classified as luminal B; cases that were ER−, PR−, and HER2+ were classified as HER2 enriched; and cases that were negative for ER, PR, and HER2 and positive for CK 5/6 and/or EGFR were categorized as basal-like. Cases that lacked expression of all 5 markers were considered “unclassified” (18).

### Assessment of Covariates

Height at baseline and recalled body weight at age 18 years were used to derive body mass index (BMI) in kg/m^2^. Weight change was estimated as the difference between current body weight and weight at age 18 years (19). Race or ethnicity was self-reported at study baselines. Family history of breast cancer in a mother or sister, personal history of benign breast disease, menopausal status, and use of oral contraceptives and/or hormone therapy (HT) was detailed at baseline and updated biennially. Women were considered postmenopausal if they reported no menstrual cycles in the last 12 months, surgical menopause with bilateral oophorectomy, or age older than 54 years for smokers or 56 years for nonsmokers. Reproductive history, including age at menarche, age at first birth, number of pregnancies lasting 6 months and over, and duration of breastfeeding, was captured at baseline in NHS and NHS II. These were updated biennially until the majority of the cohort were past reproductive age. Diet and lifestyle factors were updated every 2-4 years, including alcohol consumption, smoking status, and physical activity. We derived individuals’ adherence to the 2010 Alterative Healthy Eating Index dietary pattern with alcohol intake modeled separately, reflecting intakes of healthful and unhealthful foods and nutrients, with possible scores ranging from 2.5 to 87.5 (20). Total physical activity was captured as the frequency in engaging in common recreational activities and converted into total metabolic equivalent tasks-hours per week (21). Participants reporting a physician diagnosis of type 2 diabetes on the biennial questionnaire were sent a supplemental questionnaire (22). Confirmation of diagnosis was made according to the National Diabetes Data Group criteria through 1998 (23) and the American Diabetes Association criteria thereafter (24).

### Statistical Analysis

Dietary intakes of isoleucine, leucine, and valine were derived as grams per day, ln-transformed, then adjusted for total energy intake using the residual method (25). We analyzed intakes as the cumulative average of previous FFQs to reflect long-term usual diet. We calculated the age-standardized baseline characteristics of participants by quintiles (Q) of total BCAA intake (grams per day).

We performed Cox proportional hazards regression models to estimate the associations between quintiles of total and individual BCAAs with incident breast cancer risk. The bottom quintile (lowest intake) served as the reference group. We used quintiles for analysis a priori to examine the dose-response relationship and visualize potential for nonlinear associations. Categories also constrain influences of outlier data. Follow-up time began at the return of the first eligible FFQ until incident breast cancer diagnosis, death, or date of the last questionnaire returned through end of follow-up (NHS: June 1, 2014; NHS II: June 1, 2015), whichever came first. Cohort data were pooled for the combined analyses. In the multivariable models, age was the underlying time scale, stratified by calendar year and cohort, and we adjusted for cancer risk factors, including height (continuous), race and ethnicity (non-Hispanic White vs other), BMI at age 18 years (<20.0, 20.0-21.9, 22.0-23.9, 24.0-26.9, 27.0+ kg/m^2^), body weight change since age 18 years (continuous, kg), family history of breast cancer (yes, no), history of benign breast disease (yes, no), oral contraceptive use (never, past, current), age at menarche (<12 years, 12 years, 13 years, 14+ years), menopausal status and HT use (premenopausal, postmenopausal or unknown—never HT use, postmenopausal or unknown—past HT use, postmenopausal or unknown—current HT use, postmenopausal or unknown—missing HT use, missing both menopause status and HT use), age at natural menopause (continuous), parity and age at first birth (nulliparous, ≤2 and <25.0 years, ≤2 and 25.0-29.9 years, ≤2 and ≥30.0 years, 3+ and <25.0 years, 3+ and 25.0-29.9 years, 3+ and ≥30.0 years), total duration of breastfeeding (0, 1-6, 7-12, ≥12 months), alcohol consumption (0, <5, 5-15, 15+ g/d), smoking status (never, past, current 1-14, current 15-24, current 25+ cigarettes per day), total physical activity (metabolic equivalent task-hours/week), total energy intake (continuous, kcal/d), and Alternative Health Eating Index dietary pattern score 2010 diet quality score (quartiles). We created missing indicator categories for missing covariate data (≤5.5%). We modeled the medians of BCAA quintiles as a continuous variable to examine linear trends in the relationship of BCAAs and breast cancer.

We conducted subgroup analyses by type 2 diabetes (yes, no), premenopausal vs postmenopausal, and BMI category of normal (BMI < 25.0 kg/m^2^) vs overweight or obesity (BMI ≥ 25 kg/m^2^) to evaluate effect modification by these breast cancer risk factors. Statuses for these characteristics were updated over follow-up. We hypothesized that BCAAs may confer an elevated risk of breast cancer through those susceptible to insulin resistance and type 2 diabetes; thus, BCAAs may be positively associated among those with type 2 diabetes or obesity. We created interaction terms between median scores of BCAA quintiles and effect modifiers and used likelihood ratio tests to evaluate heterogeneity.

We performed sensitivity analyses modifying the exposure assumptions by analyzing baseline intakes only and by using only the most recent FFQ with a simple update. In exploratory analyses, we performed competing risk models to evaluate the relationships for BCAAs with tumor receptor subtypes and by menopausal status at diagnosis (26). Heterogeneity was assessed using a likelihood ratio test; we compared models assuming the same association between the exposures and breast cancer subtypes to one allowing different associations for disease subtypes.

All statistical tests were 2-sided, and a *P* less than .05 was considered statistically significant.

## Results

Our analyses included a total of 196 161 women without a history of cancer at the first valid FFQ diet assessment, including 90 154 NHS and 106 007 NHS II participants. We excluded 41 969 for the following: reporting a prior cancer, implausible estimated total energy intake (<500 kcal/d or >3500 kcal/d), and/or greater than 70 FFQ items left blank (Supplementary Figure 1, available online). The distributions of energy-adjusted BCAA intakes were similar between the NHS and the younger NHS II counterparts. Pooled age-adjusted baseline characteristics of NHS and NHS II participants by intake of total BCAAs are presented in Table 1. On average, women reporting higher intakes of BCAAs had greater body weight gain since age 18 years (Q5 = 25.7 lb vs Q1 = 21.9 lb), younger ages at menarche, and were more likely to breastfeed. We did not observe differences for race or ethnicity, family history of breast cancer, parity, and other reproductive characteristics across intakes of BCAAs. There were statistically significant differences in dietary factors across BCAA intake; higher BCAAs were associated with greater percent kcal/d from total protein (Q1 = 14% vs Q5 = 24% kcal/d) and carbohydrates (Q1 = 54% vs Q5 = 44% kcal/d), but not fat (Q1 = 32% vs Q5 = 33% kcal/d); higher animal and dairy protein intakes were positively associated with higher BCAAs, but vegetable protein intake was not related to BCAAs; overall dietary quality (Alternative Health Eating Index dietary pattern score) and regular multivitamin use were also positively associated with BCAAs.

**Table 1.** Age-standardized characteristics of the pooled study populations, NHS and NHS II, at baseline by dietary intake of BCAAs

Baseline characteristics^a^^,^^b^	Total BCAA dietary intake, g/d				
	Q1 (n = 34 884)	Q2 (n = 34 927)	Q3 (n = 34 967)	Q4 (n = 34 927)	Q5 (n = 34 903)
Mean age (SD), y	42.7 (9.3)	42.5 (9.2)	42.6 (9.2)	42.7 (9.3)	43.0 (9.4)
Mean body mass index at age 18 y (SD), kg/m^2^	20.9 (3.0)	21.0 (3.0)	21.2 (3.1)	21.4 (3.2)	22.0 (3.5)
Mean current body mass index (SD), kg/m^2^	24.0 (4.9)	24.4 (4.9)	24.7 (5.0)	25.1 (5.1)	25.8 (5.4)
Mean weight change since age 18 y (SD), lb	21.9 (31.6)	22.7 (31.0)	23.4 (31.0)	24.7 (32.4)	25.7 (34.4)
Mean height (SD), inches	64.7 (2.9)	64.7 (3.0)	64.7 (2.8)	64.7 (3.1)	64.7 (2.9)
White race or ethnicity, %	92.2	93.7	93.7	93.9	92.8
Mother or sister with breast cancer, %	8.5	8.4	8.4	8.2	8.5
Parity, No. of pregnancies ≥6 mo (SD)	2.1 (1.6)	2.2 (1.6)	2.2 (1.5)	2.2 (1.6)	2.2 (1.6)
Mean age at first full-term pregnancy (SD), y	25.4 (3.8)	25.4 (3.7)	25.5 (3.8)	25.5 (3.8)	25.5 (3.8)
Breastfeeding duration^c^, %					
Never	43.7	38.6	36.6	36.1	37.8
≤6 mo	20.9	21.6	21.8	21.9	21.3
7-11 mo	9.2	10.5	11.0	11.1	10.9
≥12 mo	19.7	23.2	24.5	24.6	23.2
Not reported	6.5	6.1	6.1	6.2	6.8
History of confirmed benign breast disease, %	32.2	32.0	32.5	32.3	32.6
Oral contraceptive use, %					
Never	32.5	31.8	31.6	31.2	31.0
Past	60.2	61.8	62.2	63.0	63.1
Current	6.7	5.9	5.8	5.3	5.4
Missing	0.6	0.5	0.4	0.5	0.6
Age at menarche, %					
≤11 y	20.8	22.4	23.2	24.0	27.5
12 y	27.9	27.9	28.4	28.8	29.0
13 y	29.8	29.7	29.6	28.8	26.4
≥14 y	21.0	19.5	18.3	18.0	16.5
Not reported	0.5	0.5	0.5	0.5	0.6
Menopausal status or hormone therapy, %					
Premenopausal	72.2	72.8	72.8	72.6	72.0
Postmenopausal and never use	13.6	13.2	13.1	12.7	12.7
Postmenopausal and past use	7.1	7.1	7.4	7.8	7.9
Postmenopausal and current use	5.9	5.7	5.6	5.8	6.2
Postmenopausal and unknown use	1.1	1.1	1.1	0.9	1.1
Not reported	0.1	0.1	0.1	0.1	0.1
Mean age at menopause (SD), y^c^	46.8 (5.9)	47.1 (5.8)	47.0 (5.9)	47.0 (5.9)	46.8 (5.9)
Mean physical activity (SD), MET, h/wk^a^	17.2 (26.2)	17.2 (24.4)	17.5 (23.0)	18.0 (23.9)	20.0 (27.1)
Current smoking status, %					
Never	53.2	55.9	55.8	56.4	55.6
Past	24.3	25.8	26.8	27.6	28.5
Current: 1-14 cigarettes/d	7.3	6.5	6.2	5.8	6.2
Current: 15-24 cigarettes/d	9.1	7.2	7.0	6.4	5.9
Current: ≥25 cigarettes/d	6.0	4.3	4.1	3.7	3.6
Not reported	0.2	0.2	0.2	0.2	0.2
Mean current alcohol intake (SD), g/d	6.7 (12.6)	5.4 (9.5)	4.8 (8.4)	4.1 (7.0)	3.1 (5.7)
AHEI-2010 diet quality score (SD)	40.2 (10.5)	41.4 (10.0)	42.2 (9.9)	43.7 (9.9)	46.9 (10.1)
Multivitamin use^b^, %	42.3	44.1	44.7	45.6	47.2
Total energy intake mean (SD), kcal/d	1754 (575)	1783 (547)	1793 (535)	1784 (523)	1725 (520)
Carbohydrates mean (SD), % kcal/d	54 (9)	50 (7)	48 (7)	46 (7)	44 (7)
Fat mean (SD), % kcal/d	32 (6)	33 (6)	34 (6)	34 (6)	33 (6)
Protein mean (SD), % kcal/d	14 (2)	17 (1)	19 (1)	20 (1)	24 (3)
Total protein mean (SD), g/d	62 (20)	74 (21)	82 (23)	89 (24)	100 (29)
Dairy protein	12 (7)	15 (8)	17 (9)	19 (11)	22 (12)
Animal protein	40 (15)	52 (15)	60 (17)	67 (18)	80 (24)
Vegetable protein	22 (9)	22 (8)	22 (8)	22 (8)	20 (8)
Isoleucine mean (SD), g/d	3 (1)	3 (1)	4 (1)	4 (1)	5 (1)
Leucine mean (SD), g/d	5 (2)	6 (2)	6 (2)	7 (2)	8 (2)
Valine mean (SD), g/d	3 (1)	4 (1)	4 (1)	5 (1)	5 (2)

^a^ Values except age are standardized to the age distribution of the study population. AHEI = Alternative Health Eating Index dietary pattern score; BCAAs = branched chain amino acids; MET = metabolic equivalent tasks; NHS = Nurses’ Health Study; Q = quintile.

^b^ Values of polytomous variables may not sum to 100% due to rounding.

c Includes natural menopause and bilateral oophorectomy.

We observed 10 046 incident breast cancer cases (NHS, n = 6621; NHS II, n = 3425) over 20.8 years median follow-up (3 644 137 person-years); 85.5% of these cases had data available for tumor ER status (ER+, n = 7005; ER−, n = 1583). Tumor tissue samples were available from 3231 breast cancer cases (NHS, n = 2399; NHS II, n = 832). Of the invasive tumors on tissue microarrays, 2858 could be classified into the luminal A (n = 1503; 52.6%), luminal B (n = 822; 28.8%), HER2-enriched (n = 178; 6.2%), basal-like (n = 283; 9.9%), or unclassified (n = 72; 2.5%) subtypes. Unclassified tumors were excluded from further analyses given the relatively small number of cases.

In the age-adjusted model, there was no relationship between the long-term cumulative average of total dietary BCAA intake with breast cancer risk, which persisted in the multivariable-adjusted model. In Table 2, the pooled cohort hazard ratio comparing the highest with lowest quintiles of intake was 1.02 (95% CI = 0.96 to 1.09, *P*_trend_ = .66) for the age-adjusted model and 1.05 (95% CI = 0.98 to 1.12, *P*_trend_ = .20) for the multivariable-adjusted model. Associations were similar for the individual BCAAs, with the multivariable models indicating no association with breast cancer risk comparing the 5th and 1st quintiles for isoleucine (hazard ratio [HR] = 1.03, 95% CI = 0.97 to 1.10, *P*_trend_ = .25), leucine HR = 1.04 (95% CI = 0.97 to 1.11, *P*_trend_ = .20), and valine HR = 1.04 (95% CI = 0.97 to 1.11, *P*_trend_ = .23). These findings were consistent between NHS and NHS II cohorts.

**Table 2.** Hazard ratios and 95% confidence intervals for the relationship between cumulative-average energy-adjusted dietary intake of BCAAs and incident invasive breast cancer risk: NHS (1984-2012) and NHS II (1991-2013)

Dietary BCAAs	Cumulative average dietary intake of energy-adjusted BCAAs, g/d					*P*_trend_
	Q1	Q2	Q3	Q4	Q5	
Total BCAAs						
Pooled						
Cases (person-years)	1961 (713 046)	2053 (752 849)	2123 (752 946)	1988 (741 234)	1921 (684 061)	
Age-adjusted model, HR (95% CI)^a^	1.00 (reference)	1.00 (0.94 to 1.06)	1.03 (0.97 to 1.10)	0.98 (0.92 to 1.04)	1.02 (0.96 to 1.09)	.66
Multivariable model, HR (95% CI)	1.00 (reference)	0.99 (0.93 to 1.05)	1.03 (0.97 to 1.10)	0.99 (0.93 to 1.05)	1.05 (0.98 to 1.12)	.20
NHS						
Cases (person-years)	1253 (344 588)	1364 (369 439)	1416 (369 836)	1307 (365 786)	1281 (337 945)	
Age-adjusted model, HR (95% CI)	1.00 (reference)	1.03 (0.95 to 1.11)	1.07 (0.99 to 1.16)	1.00 (0.92 to 1.08)	1.06 (0.98 to 1.14)	.37
Multivariable model, HR (95% CI)	1.00 (reference)	1.02 (0.94 to 1.10)	1.06 (0.98 to 1.15)	0.99 (0.92 to 1.07)	1.06 (0.98 to 1.15)	.29
NHS II						
Cases (person-years)	708 (368 459)	689 (383 410)	707 (383 111)	681 (375 448)	640 (346 116)	
Age-adjusted model, HR (95% CI)	1.00 (reference)	0.93 (0.84 to 1.04)	0.97 (0.87 to 1.07)	0.95 (0.86 to 1.06)	0.97 (0.87 to 1.08)	.71
Multivariable model, HR (95% CI)	1.00 (reference)	0.93 (0.84 to 1.04)	0.98 (0.88 to 1.09)	0.98 (0.88 to 1.09)	1.02 (0.91 to 1.14)	.57
Isoleucine						
Pooled						
Cases (person-years)	2013 (722 587)	2036 (758 521)	2153 (756 260)	1987 (739 150)	1857 (667 620)	
Age-adjusted model, HR (95% CI)	1.00 (reference)	0.97 (0.91 to 1.03)	1.03 (0.97 to 1.10)	0.97 (0.92 to 1.04)	1.01 (0.95 to 1.07)	.74
Multivariable model, HR (95% CI)	1.00 (reference)	0.96 (0.90 to 1.02)	1.03 (0.97 to 1.09)	0.98 (0.92 to 1.04)	1.03 (0.97 to 1.10)	.25
NHS						
Cases (person-years)	1286 (349 181)	1360 (371 742)	1421 (372 167)	1317 (364 802)	1237 (329 701)	
Age-adjusted model, HR (95% CI)	1.00 (reference)	1.01 (0.93 to 1.09)	1.05 (0.98 to 1.14)	1.00 (0.92 to 1.08)	1.03 (0.95 to 1.12)	.53
Multivariable model, HR (95% CI)	1.00 (reference)	1.00 (0.92 to 1.08)	1.04 (0.97 to 1.13)	0.99 (0.91 to 1.07)	1.04 (0.96 to 1.13)	.44
NHS II						
Cases (person-years)	727 (373 406)	676 (386 778)	732 (384 093)	670 (374 348)	620 (337 918)	
Age-adjusted model, HR (95% CI)	1.00 (reference)	0.90 (0.81 to 1.00)	0.99 (0.89 to 1.10)	0.94 (0.84 to 1.04)	0.96 (0.87 to 1.07)	.81
Multivariable model, HR (95% CI)	1.00 (reference)	0.90 (0.81 to 1.00)	1.00 (0.90 to 1.11)	0.96 (0.86 to 1.07)	1.01 (0.90 to 1.13)	.49
Leucine						
Pooled						
Cases (person-years)	1965 (709 581)	2032 (749 916)	2110 (750 782)	1997 (742 097)	1942 (691 761)	
Age-adjusted model, HR (95% CI)	1.00 (reference)	0.98 (0.92 to 1.04)	1.02 (0.96 to 1.09)	0.98 (0.92 to 1.04)	1.02 (0.95 to 1.08)	.66
Multivariable model, HR (95% CI)	1.00 (reference)	0.97 (0.91 to 1.04)	1.02 (0.96 to 1.09)	0.98 (0.92 to 1.05)	1.04 (0.97 to 1.11)	.20
NHS						
Cases (person-years)	1262 (343 177)	1344 (368 057)	1417 (368 907)	1308 (366 087)	1290 (341 366)	
Age-adjusted model, HR (95% CI)	1.00 (reference)	1.01 (0.93 to 1.09)	1.06 (0.98 to 1.15)	0.99 (0.91 to 1.06)	1.04 (0.96 to 1.13)	.43
Multivariable model, HR (95% CI)	1.00 (reference)	0.99 (0.92 to 1.07)	1.05 (0.97 to 1.14)	0.98 (0.91 to 1.06)	1.05 (0.96 to 1.13)	.34
NHS II						
Cases (person-years)	703 (366 404)	688 (381 860)	693 (381 875)	689 (376 010)	652 (350 395)	
Age-adjusted model, HR (95% CI)	1.00 (reference)	0.94 (0.84 to 1.04)	0.95 (0.85 to 1.05)	0.96 (0.86 to 1.07)	0.97 (0.87 to 1.08)	.79
Multivariable model, HR (95% CI)	1.00 (reference)	0.94 (0.84 to 1.04)	0.96 (0.86 to 1.07)	0.99 (0.88 to 1.10)	1.02 (0.91 to 1.14)	.50
Valine						
Pooled						
Cases (person-years)	1963 (710 566)	2040 (750 892)	2093 (752 437)	2029 (742 815)	1921 (687 427)	
Age-adjusted model, HR (95% CI)	1.00 (reference)	0.99 (0.93 to 1.05)	1.01 (0.95 to 1.08)	0.99 (0.93 to 1.05)	1.01 (0.95 to 1.08)	.75
Multivariable model, HR (95% CI)	1.00 (reference)	0.98 (0.92 to 1.04)	1.01 (0.95 to 1.08)	1.00 (0.94 to 1.06)	1.04 (0.97 to 1.11)	.23
NHS						
Cases (person-years)	1254 (343 081)	1356 (368 380)	1395 (369 563)	1337 (366 792)	1279 (339 779)	
Age-adjusted model, HR (95% CI)	1.00 (reference)	1.02 (0.94 to 1.10)	1.05 (0.97 to 1.13)	1.01 (0.93 to 1.09)	1.04 (0.96 to 1.12)	.45
Multivariable model, HR (95% CI)	1.00 (reference)	1.01 (0.93 to 1.09)	1.04 (0.96 to 1.13)	1.01 (0.93 to 1.09)	1.05 (0.96 to 1.14)	.33
NHS II						
Cases (person-years)	709 (367 485)	684 (382 512)	698 (382 874)	692 (376 023)	642 (347 649)	
Age-adjusted model	1.00 (reference)	0.92 (0.83 to 1.03)	0.95 (0.85 to 1.05)	0.96 (0.86 to 1.07)	0.96 (0.86 to 1.07)	.67
Multivariable model	1.00 (reference)	0.93 (0.83 to 1.03)	0.96 (0.86 to 1.07)	0.98 (0.88 to 1.09)	1.01 (0.90 to 1.13)	.60

^a^ Estimates were derived from Cox proportional hazards regression models adjusting for age (continuous), and additional multivariable adjustment for height (continuous), race and ethnicity (non-Hispanic White vs other), BMI at age 18 years (<20.0, 20.0-21.9, 22.0-23.9, 24.0-26.9, 27.0+ kg/m^2^), body weight change since age 18 years (continuous, kg), family history of breast cancer (yes/no), history of benign breast disease (yes, no), oral contraceptive use (never, past, current), age at menarche (<12 years, 12 years, 13 years, 14+ years), menopausal status and hormone therapy (HT) use (premenopausal, postmenopausal or unknown—never HT use, postmenopausal or unknown—past HT use, postmenopausal or unknown—current HT use, postmenopausal or unknown—missing HT use, missing both menopause status, and HT use), age at natural menopause (continuous), parity and age at first birth (nulliparous, ≤2 and <25.0 years, ≤2 and 25.0-29.9 years, ≤2 and ≥30.0 years, 3+ and <25.0 years, 3+ and 25.0-29.9 years, 3+ and ≥30.0 years), total breastfeeding (0, 1-6, 7-12, ≥12 months), alcohol consumption (0, <5, 5-15, 15+ g/d), smoking status (never, past, current 1-14, current 15-24, current 25+ cigarettes per day), total physical activity (continuous, kcal/d), and AHEI 2010 diet quality score (quartiles). AHEI = Alternative Health Eating Index dietary pattern score; BCAAs = branched chain amino acids; BMI = body mass index; HR = hazard ratio; NHS = Nurses’ Health Study.

We conducted secondary analyses to examine BCAA intake by breast cancer molecular subtypes. There was no statistically significant association between intakes of total or individual BCAAs according to subtypes of estrogen or progesterone receptor status, luminal B, basal-like, or HER2-enriched cases (Table 3). There was a modest, positive, linear trend between dietary leucine intake, but not other BCAAs, with luminal A breast cancer incidence (Q5 vs Q1 HR = 1.22, 95% CI = 1.03 to 1.45, *P*_trend_ = .03). Although trends were not statistically significant in other subtypes, the point estimates were generally similar across luminal B, HER2, and basal-like tumors.

**Table 3.** Relationship between dietary intakes of BCAAs and invasive breast cancer in the pooled NHS and NHS II by tumor receptor subtypes

Tumor receptor subtype	Cumulative average dietary intake of energy-adjusted BCAAs (g/d)					*P*_trend_
	Q1	Q2	Q3	Q4	Q5	
ER+						
Total BCAAs						
Cases (person-years)	1361 (713 620)	1457 (753 436)	1469 (753 569)	1383 (741 817)	1335 (684 615)	
Multivariable model, HR (95% CI)^a^		1.00 (0.93 to 1.08)	1.02 (0.95 to 1.10)	0.98 (0.91 to 1.06)	1.04 (0.96 to 1.12)	.59
Isoleucine						
Cases (person-years)	1406 (723 175)	1440 (759 106)	1483 (756 888)	1383 (739 735)	1293 (668 154)	
Multivariable model, HR (95% CI)		0.97 (0.90 to 1.04)	1.00 (0.93 to 1.08)	0.97 (0.89 to 1.04)	1.02 (0.94 to 1.10)	.73
Leucine						
Cases (person-years)	1364 (710 153)	1437 (750 504)	1473 (751 382)	1373 (742 711)	1358 (692 306)	
Multivariable model, HR (95% CI)		0.98 (0.91 to 1.06)	1.02 (0.94 to 1.10)	0.96 (0.89 to 1.04)	1.04 (0.96 to 1.12)	.52
Valine						
Cases (person-years)	1373 (711 134)	1438 (751 477)	1443 (753 063)	1421 (743 404)	1330 (687 980)	
Multivariable model, HR (95% CI)		0.98 (0.91 to 1.06)	0.99 (0.92 to 1.07)	0.99 (0.92 to 1.07)	1.01 (0.94 to 1.10)	.78
ER−						
Total BCAAs						
Cases (person-years)	299 (714 659)	319 (754 464)	367 (754 643)	310 (742 831)	288 (685 605)	
Multivariable model, HR (95% CI)		1.00 (0.86 to 1.18)	1.17 (1.00 to 1.36)	1.01 (0.86 to 1.19)	1.04 (0.88 to 1.23)	.59
Isoleucine						
Cases (person-years)	308 (724 242)	314 (760 134)	370 (757 970)	314 (740 757)	277 (669 099)	
Multivariable model, HR (95% CI)		0.96 (0.82 to 1.13)	1.15 (0.99 to 1.34)	1.01 (0.86 to 1.18)	1.01 (0.85 to 1.20)	.67
Leucine						
Cases (person-years)	305 (711 181)	316 (751 519)	350 (752 473)	327 (743 709)	285 (693 320)	
Multivariable model, HR (95% CI)		0.97 (0.83 to 1.14)	1.09 (0.93 to 1.27)	1.04 (0.88 to 1.22)	0.99 (0.84 to 1.17)	.78
Valine						
Cases (person-years)	293 (712 190)	323 (752 479)	361 (754 109)	316 (744 451)	290 (688 972)	
Multivariable model, HR (95% CI)		1.04 (0.88 to 1.21)	1.17 (1.00 to 1.37)	1.05 (0.89 to 1.23)	1.07 (0.90 to 1.26)	.41
ER+PR+						
Total BCAAs						
Cases (person-years)	1100 (713 865)	1206 (753 675)	1201 (753 833)	1153 (742 035)	1098 (684 832)	
Multivariable model, HR (95% CI)		1.03 (0.94 to 1.11)	1.03 (0.95 to 1.12)	1.01 (0.92 to 1.10)	1.06 (0.97 to 1.15)	.38
Isoleucine						
Cases (person-years)	1136 (723 429)	1188 (759 343)	1219 (757 147)	1151 (739 953)	1064 (668 367)	
Multivariable model, HR (95% CI)		0.99 (0.91 to 1.07)	1.02 (0.94 to 1.11)	0.99 (0.91 to 1.08)	1.04 (0.95 to 1.13)	.45
Leucine						
Cases (person-years)	1104 (710 394)	1185 (750 745)	1202 (751 653)	1149 (742 923)	1118 (692 525)	
Multivariable model, HR (95% CI)		1.00 (0.92 to 1.09)	1.02 (0.94 to 1.11)	0.99 (0.91 to 1.08)	1.05 (0.97 to 1.15)	.32
Valine						
Cases (person-years)	1109 (711 383)	1190 (751 709)	1181 (753 323)	1184 (743 624)	1094 (688 201)	
Multivariable model, HR (95% CI)		1.00 (0.92 to 1.09)	1.00 (0.92 to 1.09)	1.02 (0.93 to 1.11)	1.03 (0.95 to 1.13)	.49
ER+/PR−						
Total BCAAs						
Cases (person-years)	228 (714 730)	231 (754 555)	233 (754 760)	207 (742 922)	214 (685 679)	
Multivariable model, HR (95% CI)		0.95 (0.79 to 1.15)	0.99 (0.82 to 1.19)	0.89 (0.74 to 1.08)	1.00 (0.83 to 1.22)	.80
Isoleucine						
Cases (person-years)	235 (724 318)	233 (760 216)	231 (758 094)	208 (740 851)	206 (669 166)	
Multivariable model, HR (95% CI)		0.94 (0.78 to 1.13)	0.95 (0.79 to 1.15)	0.89 (0.73 to 1.07)	0.97 (0.80 to 1.19)	.65
Leucine						
Cases (person-years)	227 (711 260)	232 (751 608)	237 (752 557)	200 (743 836)	217 (693 385)	
Multivariable model, HR (95% CI)		0.96 (0.80 to 1.15)	1.01 (0.83 to 1.21)	0.86 (0.71 to 1.05)	1.01 (0.83 to 1.23)	.81
Valine						
Cases (person-years)	230 (712 253)	228 (752 577)	230 (754 229)	213 (744 546)	212 (689 040)	
Multivariable model, HR (95% CI)		0.93 (0.77 to 1.12)	0.96 (0.80 to 1.15)	0.90 (0.75 to 1.09)	0.97 (0.79 to 1.17)	.65
ER−/PR−						
Total BCAAs						
Cases (person-years)	259 (714 702)	289 (754 494)	319 (754 689)	276 (742 861)	261 (685 635)	
Multivariable model, HR (95% CI)		1.06 (0.90 to 1.26)	1.18 (1.00 to 1.39)	1.05 (0.88 to 1.25)	1.10 (0.92 to 1.32)	.31
Isoleucine						
Cases (person-years)	268 (724 285)	281 (760 168)	327 (758 009)	276 (740 792)	252 (669 127)	
Multivariable model, HR (95% CI)		1.00 (0.85 to 1.19)	1.18 (1.00 to 1.39)	1.03 (0.86 to 1.22)	1.07 (0.90 to 1.29)	.36
Leucine						
Cases (person-years)	265 (711 223)	286 (751 548)	306 (752 513)	290 (743 746)	257 (693 350)	
Multivariable model, HR (95% CI)		1.02 (0.86 to 1.21)	1.10 (0.93 to 1.30)	1.07 (0.90 to 1.27)	1.04 (0.87 to 1.24)	.52
Valine						
Cases (person-years)	253 (712 234)	292 (752 509)	314 (754 156)	284 (744 479)	261 (689 003)	
Multivariable model, HR (95% CI)		1.10 (0.92 to 1.30)	1.19 (1.00 to 1.40)	1.10 (0.92 to 1.31)	1.13 (0.94 to 1.35)	.20
Luminal A						
Total BCAAs						
Cases (person-years)	266 (714 692)	306 (754 518)	316 (754 689)	311 (742 824)	304 (685 595)	
Multivariable model, HR (95% CI)		1.08 (0.91 to 1.27)	1.13 (0.95 to 1.33)	1.12 (0.95 to 1.33)	1.20 (1.01 to 1.42)	.05
Isoleucine						
Cases (person-years)	275 (724 278)	287 (760 194)	328 (758 013)	314 (740 745)	299 (669 088)	
Multivariable model, HR (95% CI)		0.97 (0.82 to 1.15)	1.13 (0.96 to 1.33)	1.09 (0.93 to 1.29)	1.14 (0.96 to 1.35)	.06
Leucine						
Cases (person-years)	266 (711 222)	306 (751 570)	320 (752 489)	303 (743 735)	308 (693 302)	
Multivariable model, HR (95% CI)		1.08 (0.91 to 1.27)	1.15 (0.97 to 1.35)	1.10 (0.93 to 1.30)	1.22 (1.03 to 1.45)	.03
Valine						
Cases (person-years)	269 (712 213)	299 (752 545)	319 (754 154)	311 (744 448)	305 (688 958)	
Multivariable model, HR (95% CI)		1.04 (0.88 to 1.23)	1.12 (0.95 to 1.32)	1.11 (0.94 to 1.31)	1.18 (0.99 to 1.40)	.06
Luminal B						
Total BCAAs						
Cases (person-years)	154 (714 788)	175 (754 608)	167 (754 829)	154 (742 978)	172 (685 714)	
Multivariable model, HR (95% CI)		1.07 (0.86 to 1.33)	1.03 (0.83 to 1.29)	0.98 (0.78 to 1.23)	1.19 (0.94 to 1.49)	.25
Isoleucine						
Cases (person-years)	155 (724 386)	177 (760 261)	171 (758 156)	150 (740 909)	169 (669 205)	
Multivariable model, HR (95% CI)		1.08 (0.87 to 1.34)	1.05 (0.84 to 1.31)	0.96 (0.76 to 1.20)	1.17 (0.93 to 1.47)	.34
Leucine						
Cases (person-years)	153 (711 314)	175 (751 665)	166 (752 635)	154 (743 884)	174 (693 420)	
Multivariable model, HR (95% CI)		1.07 (0.86 to 1.34)	1.04 (0.83 to 1.30)	0.99 (0.79 to 1.25)	1.21 (0.96 to 1.52)	.18
Valine						
Cases (person-years)	154 (712 314)	182 (752 618)	156 (754 309)	158 (744 600)	172 (689 076)	
Multivariable model, HR (95% CI)		1.11 (0.89 to 1.38)	0.96 (0.77 to 1.21)	1.00 (0.80 to 1.26)	1.17 (0.93 to 1.48)	.30
HER2						
Total BCAAs						
Cases (person-years)	33 (714 912)	41 (754 736)	37 (754 960)	35 (743 081)	32 (685 849)	
Multivariable model, HR (95% CI)		1.22 (0.77 to 1.95)	1.14 (0.71 to 1.85)	1.08 (0.66 to 1.76)	1.16 (0.70 to 1.94)	.71
Isoleucine						
Cases (person-years)	34 (724 508)	44 (760 394)	33 (758 292)	36 (741 008)	31 (669 337)	
Multivariable model, HR (95% CI)		1.25 (0.79 to 1.97)	0.99 (0.60 to 1.61)	1.07 (0.66 to 1.74)	1.09 (0.65 to 1.81)	.95
Leucine						
Cases (person-years)	33 (711 439)	42 (751 788)	37 (752 766)	38 (743 985)	28 (693 561)	
Multivariable model, HR (95% CI)		1.26 (0.79 to 2.00)	1.15 (0.71 to 1.86)	1.16 (0.72 to 1.88)	1.02 (0.60 to 1.72)	.98
Valine						
Cases (person-years)	32 (712 441)	43 (752 750)	38 (754 426)	34 (744 707)	31 (689 216)	
Multivariable model, HR (95% CI)		1.31 (0.82 to 2.09)	1.20 (0.74 to 1.94)	1.16 (0.65 to 1.76)	1.14 (0.68 to 1.92)	.89
Basal-like						
Total BCAAs						
Cases (person-years)	55 (714 894)	53 (754 726)	56 (754 938)	62 (743 065)	57 (685 819)	
Multivariable model, HR (95% CI)		0.94 (0.64 to 1.37)	0.97 (0.67 to 1.42)	1.13 (0.78 to 1.64)	1.11 (0.76 to 1.64)	.35
Isoleucine						
Cases (person-years)	54 (724 492)	49 (760 391)	65 (758 259)	59 (740 995)	56 (669 305)	
Multivariable model, HR (95% CI)		0.89 (0.60 to 1.31)	1.15 (0.80 to 1.66)	1.10 (0.75 to 1.60)	1.14 (0.77 to 1.69)	.29
Leucine						
Cases (person-years)	57 (711 420)	52 (751 780)	54 (752 744)	64 (743 969)	56 (693 530)	
Multivariable model, HR (95% CI)		0.87 (0.59 to 1.27)	0.90 (0.62 to 1.32)	1.11 (0.77 to 1.60)	1.04 (0.71 to 1.53)	.48
Valine						
Cases (person-years)	53 (712 420)	53 (752 745)	61 (754 400)	59 (744 689)	57 (689 188)	
Multivariable model, HR (95% CI)		0.96 (0.66 to 1.41)	1.11 (0.76 to 1.61)	1.12 (0.76 to 1.64)	1.15 (0.78 to 1.71)	.31

^a^ Estimates were derived from Cox proportional hazards regression models adjusting for age (continuous), height (continuous), race and ethnicity (non-Hispanic White vs other), BMI at age 18 years (<20.0, 20.0-21.9, 22.0-23.9, 24.0-26.9, 27.0+ kg/m^2^), body weight change since age 18 years (continuous, kg), family history of breast cancer (yes/no), history of benign breast disease (yes, no), oral contraceptive use (never, past, current), age at menarche (<12, 12, 13, 14+ years), menopausal status and hormone therapy (HT) use (premenopausal, postmenopausal or unknown—never HT use, postmenopausal or unknown—past HT use, postmenopausal or unknown—current HT use, postmenopausal or unknown—missing HT use, missing both menopause status and HT use), age at natural menopause (continuous), parity and age at first birth (nulliparous, ≤2 and <25.0 years, ≤2 and 25.0-29.9 years, ≤2 and ≥30.0 years, 3+ and <25.0 years, 3+ and 25.0-29.9 years, 3+ and ≥30.0 years), total breastfeeding (0, 1-6, 7-12, ≥12 months), alcohol consumption (0, <5, 5-15, 15+ g/d), smoking status (never, past, current 1-14, current 15-24, current 25+ cigarettes per day), total physical activity (continuous, kcal/d), and AHEI 2010 diet quality score (quartile). AHEI = Alternative Health Eating Index dietary pattern score; BMI = body mass index; CI = confidence interval; HR = hazard ratio; NHS = Nurses’ Health Study.

We did not observe statistically significant effect modification according to type 2 diabetes diagnosis (*P*_interaction_ = .98), BMI above or below 25.0 kg/m^2^ (*P*_interaction_ = .97), or postmenopausal status (*P*_interaction_ = .11) (Supplementary Figure 2, available online). We performed sensitivity analyses modeling dietary intake derived at cohort baseline only or with a simple updating approach including only the most recently ascertained dietary data, and results between dietary BCAAs and total invasive breast cancer risk were unchanged.

## Discussion

We evaluated the relationship between long-term habitual dietary intakes of BCAAs with breast cancer incidence in 2 large US cohorts. No associations were observed for total or individual BCAAs with breast cancer risk overall or for most molecular subtypes. A modest positive association between dietary leucine with luminal A breast cancers warrants replication because it could be due to multiple testing.

Prior epidemiologic studies have identified obesity and insulin resistance as risk factors for breast cancer incidence and in particular for higher risk of postmenopausal breast cancer (27,28). A previous analysis in the NHS cohort reported a modest elevation in risk for women with a history of type 2 diabetes compared with no diabetes (HR = 1.17, 95% CI = 1.01 to 1.35), which was observed predominantly for ER+ breast cancer cases (HR = 1.22, 95% CI = 1.01 to 1.47) (29). BCAA metabolites in circulation have been implicated for their strong positive correlation with obesity, clinical markers of impaired carbohydrate metabolism, and incident type 2 diabetes risk, and therefore an underlying role in breast cancer development or progression is plausible. We recently reported complex findings between plasma BCAA metabolites with breast cancer risk in subsets of the NHS and NHS II cohorts (Zeleznik O, et al., unpublished data). BCAAs were prospectively associated with lower breast cancer risk among premenopausal participants but with higher breast cancer risk among postmenopausal women within 10 years from blood collection. Further evidence in support of a role for BCAAs has been demonstrated through the effects of in vitro leucine administration impact on cell proliferation and treatment resistance in ER+ breast cancer cells (15).

Isoleucine, leucine, and valine are essential amino acids and thus derived solely from diet; therefore, we sought to evaluate their upstream dietary intakes in relation to breast cancer incidence at the population level. However, we observed that despite long-term follow-up and repeated dietary assessments, dietary intakes of BCAAs were not related to breast cancer incidence in our cohorts. There are reasons that may explain these unexpected results. Firstly, although BCAAs are essential amino acids, their dietary intake may correlate only modestly with levels found in circulation. For example, in a previous analysis among women with a history of gestational diabetes, we observed correlations *r* < 0.2 comparing dietary vs circulating plasma levels of individual BCAAs (8). Further, in this analysis, whereas plasma BCAAs were positively associated with subsequent type 2 diabetes risk, dietary intakes were not. Thus, it is possible that levels in circulation correlate poorly with dietary intakes because they more closely reflect capacity for rate of BCAA metabolism (30). Determinants of variability in the rate of BCAA metabolism are largely unknown; 2 randomized intervention trials demonstrated an effect of weight loss on decreases in circulating BCAA levels (31), and an exercise training intervention similarly observed greater BCAA turnover in parallel with increased insulin sensitivity (32). This suggests a plausible role for modifiable lifestyle interventions in improving BCAA exposure in circulation. Whether modifying dietary intakes of BCAAs in the absence of interventions modifying their postprandial catabolism lowers BCAAs in circulation is unknown. Thus, although dietary BCAAs were not related to breast cancer in our cohorts, we cannot rule out an association for BCAA metabolite levels. It is also plausible that BCAAs are not causally related to breast cancer incidence, despite promising hypothesis-generating studies. Further, our ability to isolate the contribution of BCAAs independent of correlated dietary components in BCAA-containing foods is limited, and thus findings like that for leucine with luminal A cancers should be interpreted with caution.

The strengths of this study include its prospective cohort design with longitudinal assessment of diet before breast cancer diagnosis. We analyzed the cumulative average of diet reported every 4 years, which reduces measurement error for estimations of long-term intake. The performance of the FFQ has been extensively validated and reliably estimates protein intake. The race and ethnic homogeneity of the NHS and NHS II cohorts is a limitation precluding our ability to investigate potential effect modification by these important breast cancer risk factors. Misclassification of breast cancer cases is unlikely with 99% confirmation of self-reported cases.

Identifying dietary risk factors underlying the relationships of diet and body weight with cancer may inform strategies for precision prevention that efficiently target specific pathways of breast cancer in women. However, despite prior evidence implicating circulating BCAAs, we did not observe an association between dietary intakes of total and individual BCAAs with breast cancer incidence in our large cohort of predominantly White US women. Further investigation into determinants of circulating BCAA concentrations, which may better reflect long-term systemic exposure to BCAAs and impaired metabolism, is warranted.

## Funding

This study was funded by the National Cancer Institute (R01 CA050385, P01 CA087969, U01 CA176726, UM1 CA186107) and the Breast Cancer Research Foundation.

## Notes

**Role of the funders:** Funders had no role in design of the study; the collection, analysis, and interpretation of the data; the writing of the manuscript; and the decision to submit the manuscript for publication.

**Disclosures:** None.

**Author contributions**: Statistical analysis: Dt, BC, AHE; Manuscript draft: DT; Manuscript edits: DT, BC, RT, JM, FH, WW, AHE; Funding and study design: RM, JM, WW, AHE.

**Acknowledgements:** We would like to thank the participants and staff of the Nurses’ Health Studies for their valuable contributions as well as the following state cancer registries for their help: AL, AZ, AR, CA, CO, CT, DE, FL, GA, ID, IL, IN, IA, KY, LA, ME, MD, MA, MI, NE, NH, NJ, NY, NC, ND, OH, OK, OR, PA, RI, SC, TN, TX, VA, WA, WY.

**Disclaimer:** The authors assume full responsibility for analyses and interpretation of these data.

## Data Availability

The datasets analyzed during the current study are available by application by following the instructions here: http://www.nurseshealthstudy.org/researchers.

## Supplementary Material

pkab032_Supplementary_Data

## References

[1] Wu G. Functional amino acids in nutrition and health. Amino Acids. 2013;45(3):407–411. 23595206 10.1007/s00726-013-1500-6

[2] Guasch-Ferre M, Hruby A, Toledo E, et al Metabolomics in prediabetes and diabetes: a systematic review and meta-analysis. Dia Care. 2016;39(5):833–846. 10.2337/dc15-2251 PMC4839172 27208380

[3] Tobias DK, Lawler PR, Harada PH, et al Circulating branched-chain amino acids and incident cardiovascular disease in a prospective cohort of US women. Circ Genom Precis Med. 2018;11(4):e002157. 29572205 10.1161/CIRCGEN.118.002157 PMC5880282

[4] Mayers JR, Wu C, Clish CB, et al Elevation of circulating branched-chain amino acids is an early event in human pancreatic adenocarcinoma development. Nat Med. 2014;20(10):1193–1198. 25261994 10.1038/nm.3686 PMC4191991

[5] Zeleznik ZA, Balasubramanian R, Ren T, et al Branched chain amino acids and risk of breast cancer. medRxiv. 2020. doi: 10.1101/2020.08.31.20185470. PMC8460878 34585062

[6] Wu J, Zeng R, Huang J, et al Dietary protein sources and incidence of breast cancer: a dose-response meta-analysis of prospective studies. Nutrients. 2016;8(11):730. 10.3390/nu8110730 PMC5133114 27869663

[7] Farvid MS, Cho E, Chen WY, Eliassen AH, Willett WC. Dietary protein sources in early adulthood and breast cancer incidence: prospective cohort study. BMJ. 2014;348:g3437. 24916719 10.1136/bmj.g3437 PMC4051890

[8] Tobias DK, Clish C, Mora S, et al Dietary intakes and circulating concentrations of branched-chain amino acids in relation to incident type 2 diabetes risk among high-risk women with a history of gestational diabetes mellitus. Clin Chem. 2018;64(8):1203–1210. 29945965 10.1373/clinchem.2017.285841 PMC6434682

[9] Saha AK, Xu XJ, Balon TW, Brandon A, Kraegen EW, Ruderman NB. Insulin resistance due to nutrient excess: is it a consequence of AMPK downregulation? Cell Cycle. 2011;10(20):3447–3451. 22067655 10.4161/cc.10.20.17886 PMC3356833

[10] Saha AK, Xu XJ, Lawson E, et al Downregulation of AMPK accompanies leucine- and glucose-induced increases in protein synthesis and insulin resistance in rat skeletal muscle. Diabetes. 2010;59(10):2426–2434. 20682696 10.2337/db09-1870 PMC3279521

[11] Tremblay F, Marette A. Amino acid and insulin signaling via the mTOR/p70 S6 kinase pathway. A negative feedback mechanism leading to insulin resistance in skeletal muscle cells. J Biol Chem. 2001;276(41):38052–38060. 11498541 10.1074/jbc.M106703200

[12] Krebs M, Brunmair B, Brehm A, et al The mammalian target of rapamycin pathway regulates nutrient-sensitive glucose uptake in man. Diabetes. 2007;56(6):1600–1607. 17329620 10.2337/db06-1016

[13] Krebs M, Krssak M, Bernroider E, et al Mechanism of amino acid-induced skeletal muscle insulin resistance in humans. Diabetes. 2002;51(3):599–605. 11872656 10.2337/diabetes.51.3.599

[14] Tremblay F, Krebs M, Dombrowski L, et al Overactivation of S6 kinase 1 as a cause of human insulin resistance during increased amino acid availability. Diabetes. 2005;54(9):2674–2684. 16123357 10.2337/diabetes.54.9.2674

[15] Saito Y, Li L, Coyaud E, et al LLGL2 rescues nutrient stress by promoting leucine uptake in ER(+) breast cancer. Nature. 2019;569(7755):275–279. 30996345 10.1038/s41586-019-1126-2

[16] Yuan C, Spiegelman D, Rimm EB, et al Validity of a dietary questionnaire assessed by comparison with multiple weighed dietary records or 24-hour recalls. Am J Epidemiol. 2017;185(7):570–584. 28338828 10.1093/aje/kww104 PMC5859994

[17] Fung TT, Chiuve SE, Willett WC, Hankinson SE, Hu FB, Holmes MD. Intake of specific fruits and vegetables in relation to risk of estrogen receptor-negative breast cancer among postmenopausal women. Breast Cancer Res Treat. 2013;138(3):925–930. 23532538 10.1007/s10549-013-2484-3 PMC3641647

[18] Fortner RT, Sisti J, Chai B, et al Parity, breastfeeding, and breast cancer risk by hormone receptor status and molecular phenotype: results from the Nurses' Health Studies. Breast Cancer Res. 2019;21(1):40. 30867002 10.1186/s13058-019-1119-y PMC6416887

[19] Rimm EB, Stampfer MJ, Colditz GA, Chute CG, Litin LB, Willett WC. Validity of self-reported waist and hip circumferences in men and women. Epidemiology. 1990;1(6):466–473. 2090285 10.1097/00001648-199011000-00009

[20] Tobias DK, Hu FB, Chavarro J, Rosner B, Mozaffarian D, Zhang C. Healthful dietary patterns and type 2 diabetes mellitus risk among women with a history of gestational diabetes mellitus. Arch Intern Med. 2012;172(20):1566–1572. 22987062 10.1001/archinternmed.2012.3747 PMC3774782

[21] Wolf AM, Hunter DJ, Colditz GA, et al Reproducibility and validity of a self-administered physical activity questionnaire. Int J Epidemiol. 1994;23(5):991–999. 7860180 10.1093/ije/23.5.991

[22] Manson JE, Rimm EB, Stampfer MJ, et al Physical activity and incidence of non-insulin-dependent diabetes mellitus in women. Lancet. 1991;338(8770):774–778. 1681160 10.1016/0140-6736(91)90664-b

[23] National Diabetes Data Group. Classification and diagnosis of diabetes mellitus and other categories of glucose intolerance. Diabetes. 1979;28(12):1039–1057. 510803 10.2337/diab.28.12.1039

[24] Report of the Expert Committee on the Diagnosis and Classification of Diabetes Mellitus. Diabetes Care. 1997;20(7):1183–1197. 9203460 10.2337/diacare.20.7.1183

[25] Willett W, Stampfer MJ. Total energy intake: implications for epidemiologic analyses. Am J Epidemiol. 1986;124(1):17–27. 3521261 10.1093/oxfordjournals.aje.a114366

[26] Wang M, Spiegelman D, Kuchiba A, et al Statistical methods for studying disease subtype heterogeneity. Statist Med. 2016;35(5):782–800. 10.1002/sim.6793 PMC4728021 26619806

[27] Renehan AG, Tyson M, Egger M, Heller RF, Zwahlen M. Body-mass index and incidence of cancer: a systematic review and meta-analysis of prospective observational studies. Lancet. 2008;371(9612):569–578. 18280327 10.1016/S0140-6736(08)60269-X

[28] Maskarinec G, Fontaine A, Torfadottir JE, et al The relation of type 2 diabetes and breast cancer incidence in Asian, Hispanic and African American populations-a review. Can J Diabetes. 2018;42(1):100–105. 28506814 10.1016/j.jcjd.2017.02.005

[29] Michels KB, Solomon CG, Hu FB, et al; Nurses' Health Study. Type 2 diabetes and subsequent incidence of breast cancer in the Nurses' Health Study. Diabetes Care. 2003;26(6):1752–1758. 12766105 10.2337/diacare.26.6.1752

[30] Tobias DK, Mora S, Verma S, Lawler PR. Altered branched chain amino acid metabolism: toward a unifying cardiometabolic hypothesis. Curr Opin Cardiol. 2018;33(5):558–564. 29994805 10.1097/HCO.0000000000000552 PMC6260824

[31] Zheng Y, Ceglarek U, Huang T, et al Weight-loss diets and 2-y changes in circulating amino acids in 2 randomized intervention trials. Am J Clin Nutri. 2016;103(2):505–511. 10.3945/ajcn.115.117689 PMC4733257 26791187

[32] Glynn EL, Piner LW, Huffman KM, et al Impact of combined resistance and aerobic exercise training on branched-chain amino acid turnover, glycine metabolism and insulin sensitivity in overweight humans. Diabetologia. 2015;58(10):2324–2335. 26254576 10.1007/s00125-015-3705-6 PMC4793723

