# Supplementary material for: Dietary Intake of Branched Chain Amino Acids and Breast Cancer Risk in the NHS and NHS II Prospective Cohorts
Source: JNCI Cancer Spectr. 2021 Apr 12;5(3):pkab032. doi: 10.1093/jncics/pkab032 (PMC8494188; doi:10.1093/jncics/pkab032)

Supplementary Figure 1. Participants eligible for inclusion in analysis

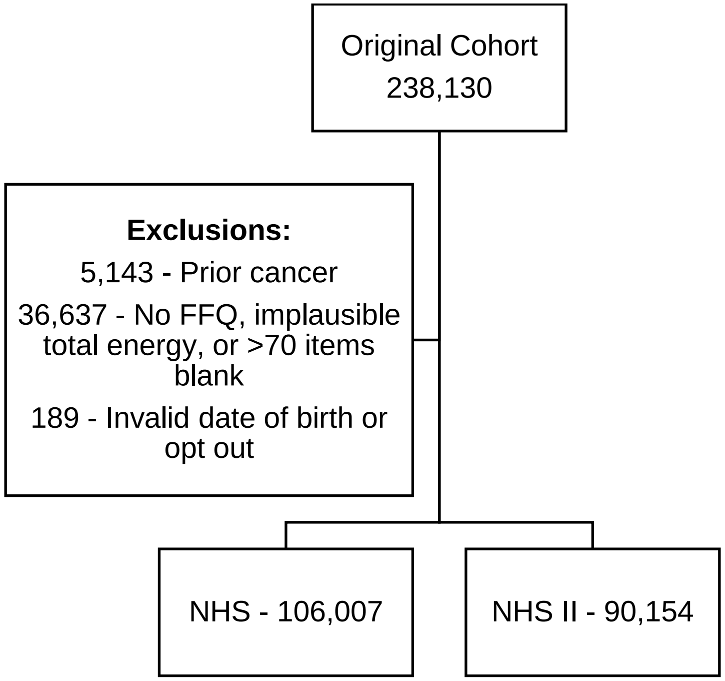

Supplementary Figure 2. Multivariable-adjusted hazards ratio and 95% CI for dietary intakes of branched-chain amino acids (g/d) and invasive breast cancer in the pooled Nurses' Health Study and Nurses' Health Study II, by subgroup

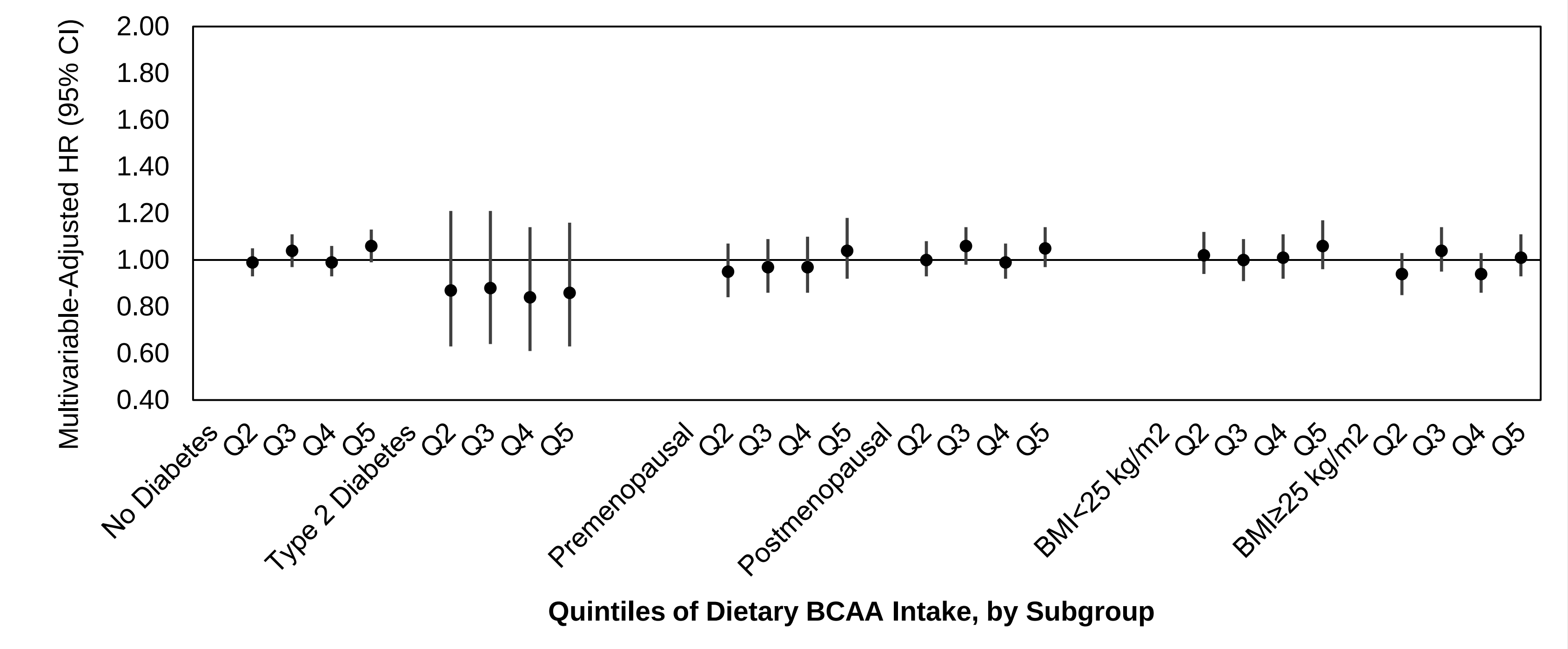

Supplement: pkab032_Supplementary_Data [file pkab032_supplementary_data.pdf]
